# Aloysia Citrodora Essential Oil Inhibits Melanoma Cell Growth and Migration by Targeting HB-EGF-EGFR Signaling

**DOI:** 10.3390/ijms22158151

**Published:** 2021-07-29

**Authors:** Yousef Salama, Nidal Jaradat, Koichi Hattori, Beate Heissig

**Affiliations:** 1 An-Najah Center for Cancer and Stem Cell Research, Faculty of Medicine and Health Sciences, An-Najah National University, P.O. Box 7, Nablus 99900800, Palestine; 2 Department of Pharmacy, Faculty of Medicine and Health Sciences, An-Najah National University, Nablus 00970, Palestine; nidaljaradat@najah.edu; 3 Center for Genomic & Regenerative Medicine, School of Medicine, Juntendo University, 2-1-1 Hongo, Bunkyo-Ku, Tokyo 113-8421, Japan; khattori@juntendo.ac.jp; 4 Department of Immunological Diagnosis, School of Medicine, Juntendo University, 2-1-1 Hongo, Bunkyo-Ku, Tokyo 113-8421, Japan

**Keywords:** melanoma, EGFR, MMP, Aloysia citrodora, HB-EGF, plant, proliferation, metastasis, ERK1/2, herb

## Abstract

Patients diagnosed with melanoma have a poor prognosis due to regional invasion and metastases. The receptor tyrosine kinase epidermal growth factor receptor (EGFR) is found in a subtype of melanoma with a poor prognosis and contributes to drug resistance. Aloysia citrodora essential oil (ALOC-EO) possesses an antitumor effect. Understanding signaling pathways that contribute to the antitumor of ALOC-EO is important to identify novel tumor types that can be targeted by ALOC-EO. Here, we investigated the effects of ALOC-EO on melanoma growth and tumor cell migration. ALOC-EO blocked melanoma growth in vitro and impaired primary tumor cell growth in vivo. Mechanistically, ALOC-EO blocked heparin-binding-epidermal growth factor (HB-EGF)-induced EGFR signaling and suppressed ERK1/2 phosphorylation. Myelosuppressive drugs upregulated HB-EGF and EGFR expression in melanoma cells. Cotreatment of myelosuppressive drugs with ALOC-EO improved the antitumor activity and inhibited the expression of matrix metalloproteinase-7 and -9 and a disintegrin and metalloproteinase domain-containing protein9. In summary, our study demonstrates that ALOC-EO blocks EGFR and ERK1/2 signaling, with preclinical efficacy as a monotherapy or in combination with myelosuppressive drugs in melanoma.

## 1. Introduction

Cutaneous melanoma is an aggressive tumor with increasing incidence worldwide. B-Raf has a mutation rate of up to 90% in melanoma [1,2,3]. Despite major efforts to improve treatment, including the introduction of signal transduction inhibitors like B-Raf enzyme inhibitor and MEK inhibitor or immune checkpoint blocker, no significant advances in patient survival have been obtained in the last two decades in patients with advanced disease [4,5,6,7]. One problem in treating patients is that tumors can develop acquired resistance. Novel drugs are urgently needed.

Essential oils (EO), which are concentrated hydrophobic liquids from aromatic plants, have shown anticancer properties enabling them to penetrate the cell membrane and act on cellular targets like Akt and mTOR, and have been shown to increase apoptosis by upregulating caspases 3 and 9 [8]. Metabolites contained within EOs help plants defend themselves against herbivores, insects, and microorganisms [9,10]. A cheap alternative to conventional chemotherapeutic drugs for overcoming the occurrence of multidrug resistance, the anticancer effects of plants are being brought back into the spotlight. Aloysia citrodora (ALOC) Paláu (Lippia citriodora Kunth), also known as “lemon verbena”, is a medicinal plant native to South America, North Africa, and southern Europe, where it is used by native people to treat diarrhea, flatulence, insomnia, and rheumatism, among other maladies [11]. The ALOC EO contains chemically aromatic secondary plant metabolites like neral, geranial, limonene, and 1,8-cineole, while the extract contains verbascoside derivates and flavonoids [11]. EOs from ALOC have been reported to show anticancer activities [12]. Studies by Zeng et al. showed that geranial and neral inhibited tumor growth in the 4T1 breast cancer xenograft mouse model [13].

Autocrine/paracrine production of epidermal growth factor receptor (EGFR) ligands, like epiregulin, amphiregulin, and heparin-binding EGF-like growth factor (HB-EGF), and the overexpression of EGFR, are two of the mechanisms most frequently implicated in cancer development and progression. HB-EGF is involved in the progression of tumors like hepatocellular carcinoma, colon carcinoma, and melanoma. HB-EGF promotes melanoma growth through its interaction with EGFR and in its role as a MAPK and PI3K/Akt pathway activator [14,15,16]. Expression of EGFR in melanoma establishes a pro-metastatic phenotype [17] that can activate Ras/MAPK, PLCγ1/PKC, Akt, and STAT, which subsequently stimulate cell proliferation, migration, invasion, survival, and differentiation. The receptor tyrosine kinase EGFR is activated in a subset of melanoma cells [18,19] where its expression correlates with poor prognosis in human melanoma [20,21]. 

The expression of various receptor tyrosine kinases, like AXL, PDGFRB, and EGFR, in cutaneous melanoma occurs in MITF^low^ melanoma cells with a pro-invasive potential and has been associated with BRAF/MEK inhibitor resistance. Studies using human melanoma cell lines and experimental murine melanoma models found that EGFRs are promising therapeutic targets. Bardeesy et al. demonstrated the existence of an EGF signaling loop essential for H-RASV12G-mediated tumorigenesis using an inducible transgenic mouse model [22]. EGFR gene copy number alterations and polysomy of chromosome 7—where the EGFR gene is located—are correlated with poorer prognosis in human melanoma [23]. In addition, EGFR expression is higher in metastases compared with primary tumors [24]. 

Metalloproteinase-7 (MMP7) expression was demonstrated in the majority of primary tumors and all metastatic tumors in melanomas and therefore was proposed as a prognostic marker [25]. MMP-9 (gelatinase B), similar to MMP-2, is present in melanoma both in tumor cells and stroma [26]. A disintegrin and metalloproteinase domain-containing protein 9 (ADAM9) is expressed in human melanoma at the tumor-stroma border, where direct or indirect interactions between tumor cells and fibroblasts occur [27]. 

In this study, we extracted ALOC-EC from the plant. ALOC-EO showed significant efficacy in a murine syngeneic melanoma model. ALOC-EC treatment downregulates HB-EGF and EGFR, and EGFR downstream target proteases. It induces apoptosis and prevents cell migration. Furthermore, it demonstrates synergy with the Food and Drug Administration (FDA)-approved antitumor drugs, doxorubicin and bortezomib, in killing melanoma cells.

## 2. Results

### 2.1. Growth Inhibition and Induction of Apoptosis by ALOC-EO in Melanoma Cells

Gas chromatography-mass spectroscopy analysis of the essential oil (EO) isolated from Aloysia citrodora (ALOC-EO) revealed the presence of 24 components with the main compounds being geranial (26.61%), neral (20.03%), D-limonene (14.84%), and α-curcumene (13.97%) (Supplementary Table S1), supporting previous reports on ALOC-EO from other demographic regions [11]. The ALOC-EO consisted mainly of oxygenated monoterpenes and hydrocarbon sesquiterpenes according to the characterization and phytochemical classification (Supplementary Table S1). 

**Figure 1:**
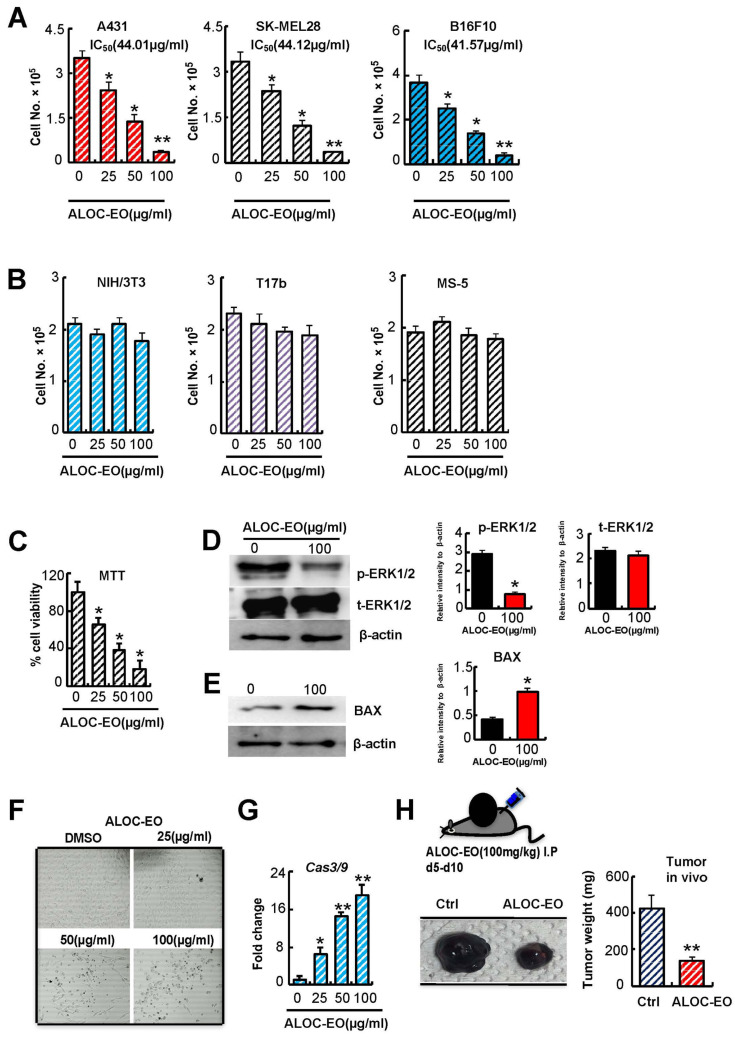
ALOC-EO is cytotoxic for melanoma cells. (**A**) The murine B16F10, human SK-MEL28, human epidermoid carcinoma cell line A-431, and (**B**) the murine fibroblast NIH/3T3, embryonic endothelial progenitor cells (T17b), or the stromal cell line MS5, were exposed to indicated concentrations of ALOC-EO for 24 h in a 5% CO_2_ incubator at 37 °C. Cells were counted using a hematocytometer (n = 6/group). The inhibitory concentration 50 (IC_50_) for each cell line is given. (**C**) Cell viability of B16F10 cells exposed to indicated concentrations of ALOC-EO was determined using an MTT assay (n = 6/group). (**D**) Total ERK1/2 and phosphorylation of ERK1/2 in B16F10 cells treated with either DMSO (control) or ALOC-EO after 4 h (100 µg/mL). β-actin served as a loading control. Left panel immunoblot. Right panel: The histograms represent the signal intensity of the indicated protein bands in arbitrary units after normalization with the signal intensity of β-actin internal control for each sample. Values are means and SEM of two independent experiments. (**E**) Representative immunoblots for BAX in untreated and ALOC-EO-treated B16F10 cell lysates. β-actin served as a loading control. Right panel: The histogram represents the signal intensity of the indicated protein bands in arbitrary units after normalization with the signal intensity of β-actin internal control for each sample. Values are means and SEM of two independent experiments. (**F**) Macroscopic images of B16F10 cells treated with/without ALOC-EC were taken using inverted microscopy (magnification 20x). (**G**) Fold change in *Caspase-3* and *Caspase-9* expression in ALOC-EO and control cells by quantitative PCR (qPCR). Transcript levels were normalized to β-actin. (**H**) B16F10 were injected s.c. into the right flanks of C57/BL6 mice and treated with or without ALOC-EO starting from day 5. Twelve days after inoculation, macroscopic tumor images were taken (left panel) and the tumor weight was measured (right panel; n = 6/group). * *p* < 0.05; ** *p* < 0.01 (Student’s *t*-test).

Several terpenoids are known for their tumor-suppressive properties of various types of cancer, such as breast, lung, colon, prostate, pancreatic, and hepatic cancer, and suppress skin carcinogenesis [28,29,30]. Here, we investigated the anticancer properties of ALOC-EO on a panel of different skin tumor cell lines, including the human epidermoid carcinoma A-431, the human melanoma SK-MEL-28, and the mouse melanoma B16F10 cell lines. We observed a significant decrease in proliferation for all ALOC-EO treated cells compared with controls in a dose-dependent manner. The half-maximal inhibitory concentrations (IC_50_) for A431, SKL28, and B16F10 were 44.01, 44.12, and 41.57 µg/mL, respectively (Figure 1A).

ALOC-EC was tested for cytotoxicity by the MTT (3-(4,5-dimethylthiazol-2-yl)-2,5-diphenyltetrazolium bromide) assay in the highly metastatic B16F10 murine melanoma cell line. Cell viability decreased in a dose-dependent manner as determined by the MTT assay (Figure 1B,C).

### 2.2. ALOC_EO Blocks Melanoma Cell Proliferation and ERK1/2 Phosphorylation

Because melanoma proliferation is dependent upon extracellular signal-regulated kinase 1/2 (ERK1/2) phosphorylation, we examined the effects of ALOC-EO on ERK1/2 phosphorylation. ALOC-EO treatment impaired ERK1/2 phosphorylation but did not increase total ERK1/2 in B16F10 cells as shown using Western blotting analysis (Figure 1D).

Macroscopically, we observed shrinkage of tumor cells and nuclei and the condensation of nuclear chromatin into sharply delineated masses and karyorrhexis (nucleus break up) in ALOC-EO treated cells, suggesting that ALOC-EO activates apoptotic pathways in treated cells (Figure 1E). Therefore, we examined the expression of apoptosis-linked factors like BAX and caspase-3 and -9. ALOC-EO induced apoptosis in treated melanoma cells with the upregulation of the intrinsic apoptotic pathway-associated BAX protein (Figure 1F). ALOC-EO increases in *caspase-3 and -9* expression as determined by quantitative PCR (qPCR) (Figure 1G). These data indicate that ALOC-EO activates the intrinsic apoptotic pathway.

To further validate the anti-proliferative and anticancer potential of ALOC-EO in vivo, ALOC-EO was administered to tumor-bearing mice in an orthotopic murine melanoma model. B16F10 cells were subcutaneously injected into C57/BL6 mice, and after small tumors were visible (by day 5), groups of mice were injected with ALOC-EO or carrier intraperitoneally (i.p.). As shown in Figure 1G, the tumor volumes of ALOC-EO-treated mice were reduced when compared to respective controls, further supporting the anticancer and anti-proliferative potential of ALOC-EO through the induction of apoptosis.

### 2.3. ALOC-EO Inhibits Tumor Cell Migration and MMP Expression

**Figure 2:**
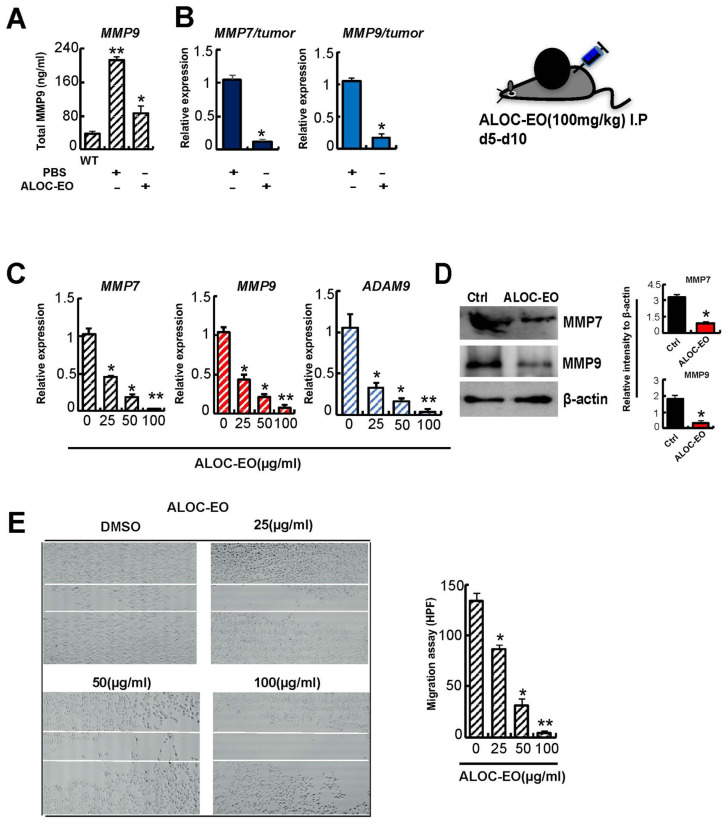
ALOC-EO blocks melanoma cell migration and MMP expression. (**A**) MMP9 levels in the plasma of B16F10-tumor-bearing mice injected with/without ALOC-EO starting from day 5 (n = 6/group). (**B**) *MMP7* and *MMP9* expression was determined by qPCR in tumor tissues of ALOC-EO treated and untreated mice. (**C**) *MMP7*, *MMP9*, and *ADAM9* expression were determined by qPCR in B16F10 cells cultured for 24 h with indicated concentrations of ALOC-EO. (**B**,**C**) Transcript levels were normalized to β-actin. Graphs represent averages from three to seven independently prepared templates. The data represent two independent experiments with similar results. (**D**) Western blot analysis of MMP7, MMP9, and the loading control β-actin in B16F10 cells treated with DMSO (control) or ALOC-EO. Right panel: The histograms represent the signal intensity of the indicated protein bands in arbitrary units after normalization with the signal intensity of β-actin internal control for each sample. Values are means and SEM of two independent experiments. (**E**) Faster wound closure in B16F10 cultures supplemented with DMSO or ALOC-EO (left panel). The migrated cells in the scraped area (right panel) were counted per high power fields (HPF). A total of six HPF per condition were evaluated. Data are mean +/− SEM. * *p* < 0.05; ** *p* < 0.01 (Student’s *t*-test).

The extracellular matrix (ECM)-degrading proteases comprising a disintegrin and metalloprotease-9 (ADAM9), matrix metalloproteinase-7 (MMP7), and MMP9 play a pivotal role during melanoma growth, migration, and adhesion [25,27]. ALOC-EO-treated, but not control B16F10 tumor-carrying mice showed lower total MMP9 plasma levels and a lower *MMP7* and *MMP9* gene expression in extracted tumors (Figure 2A,B). In addition, impaired *MMP7*, *MMP9*, and *ADAM9* expression were found in ALOC-EO-treated B16F10 cells in a dose-dependent manner in vitro (Figure 2C,D) indicating that ALOC-EO suppresses the expression of tumor cell-derived proteases.

Given the recent emphasis on the role of MMP9 and MMP7 during cancer cell migration, we next investigated the effect of ALOC-EO on the migration of B16F10 melanoma cells grown in vitro. Cells migrated into the scratched area were quantified. We found that cells treated with medium migrated freely, while ALOC-EO-treated cell migration was inhibited (Figure 2E). Although it cannot be ruled out that the migration suppressive effects of ALOC-EO were different from the apoptosis-inducing effects, we did not observe substantial cell death in ALOC-EO-treated cells during the 4 h migration period (data not shown). 

### 2.4. ALOC-EO Blocks EGFR-Mediated Melanoma Cell Growth and MMP Production

**Figure 3:**
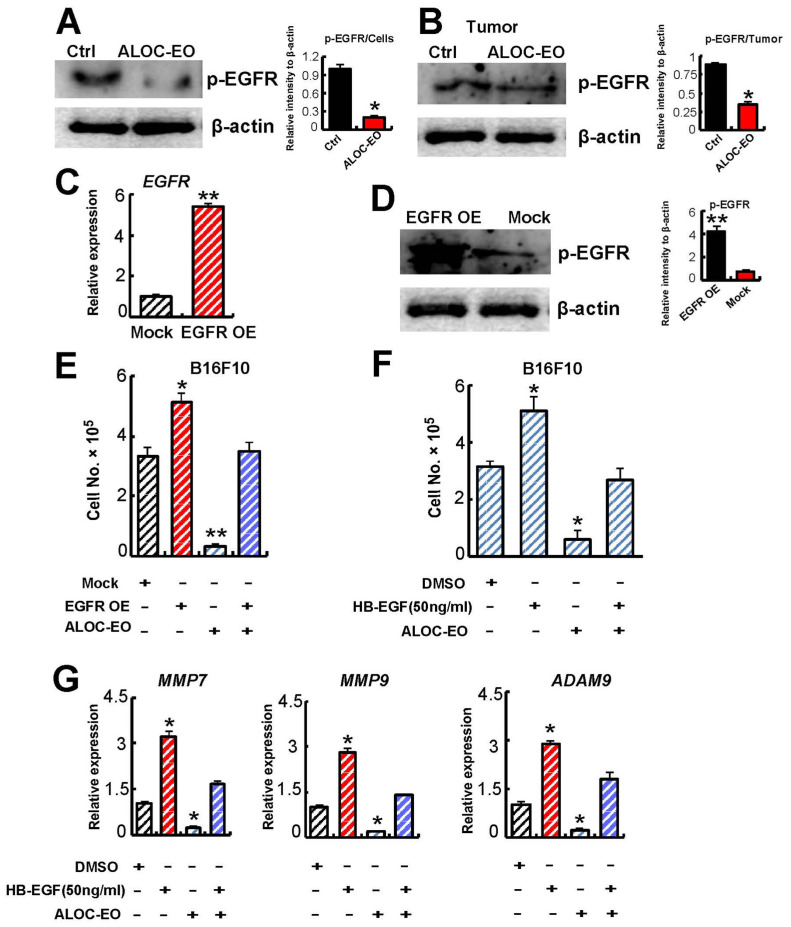
ALOC-EO targets EGFR and ERK1/2. (**A**) Immunoblot for phosphorylated murine EGFR in B16F10 cells treated with either DMSO or ALOC-EO after 12 h. Right panel: The histogram represents the signal intensity of the indicated protein bands in arbitrary units after normalization with the signal intensity of β-actin internal control for each sample. Values are means and SEM of two independent experiments. (**B**) Representative immunoblot for phosphorylated murine EGFR and loading control β-actin in tumor cell lysates from control and ALOC-EO-treated mice. Right panel: The histogram represents the signal intensity of the indicated protein bands in arbitrary units after normalization with the signal intensity of β-actin internal control for each sample. Values are means and SEM of two independent experiments. (**C**) EGFR overexpression (EGFR OE) was confirmed in B16F10 cells and mock controls by qPCR, and the fold change in *EGFR* expression compared to expression of β-actin is given (n = 3). (**D**) EGFR phosphorylation was performed in cell lysates of EGFR OE and mock cells by Western blotting. Right panel: The histogram represents the signal intensity of the indicated protein bands in arbitrary units after normalization with the signal intensity of β-actin internal control for each sample. Values are means and SEM of two independent experiments. (**E**) EGFR OE and mock cells were treated with/without ALOC-EO. Cells were counted after 24 h in culture (n = 6/group). (**F**) B16F10 cells were treated with/without rec. HB-EGF in the presence or absence of ALOC-OC. Cells were counted 24 h later (n = 6/group). (**G**) Murine *MMP7*, *MMP9*, *ADAM9*, and *HB-EGF* expression in EGFR OE and mock B16F10 cells treated with or without HB-EGF in the presence or absence of ALOC-EO by qPCR. Transcript levels were normalized to β-actin. +/− SEM. * *p* < 0.05; ** *p* < 0.01 (Student’s *t*-test).

α-Curcumene is one of the major compounds in ALOC-EO (Supplementary Figure S1A). α-Curcumin can block tumor growth by targeting epidermal growth factor receptor (EGFR) among others [31]. Previous studies showed that RAS regulates EGFR signaling [22]. RAS-directed melanoma tumor maintenance in vivo had been shown to depend on a functionally relevant EGFR autocrine loop. To examine whether ALOC-EO modulates EGFR signaling, EGFR phosphorylation was assessed after ALOC-EO stimulation. Representative immunoblot analysis confirmed that ALOC-EO suppressed EGFR phosphorylation in ALOC-EC-treated B16F10 cells in vitro (Figure 3A). Similarly, tumor tissues retrieved from ALOC-EO treated mice showed impaired EGFR phosphorylation mice (Figure 3B) indicating that ALOC-EO suppresses EGFR signaling.

To further explore the role of EGFR signaling as a target of ALOC-EO, we generated EGFR overexpressing melanoma cells (EGFR OE). EGFR overexpression was confirmed in EGFR OE cells by qPCR (Figure 3C). Melanoma and glioblastoma cell lines express HB-EGF and TGF-α, the main ligands of EGFR [32]. EGFR OE cells showed enhanced EGFR phosphorylation by Western blotting (Figure 3D). When cells were treated with HB-EGF, ALOC-EO treatment prevented HB-EGF-mediated cell proliferation (Figure 3F). 

Because EGFR signaling promotes melanoma cell proliferation, and ALOC-EO targets EGFR signaling, we assessed the proliferation-inhibitory effects of ALOC-EO on EGFR OE cells or cells treated with rec. HB-EGF. ALOC-EO prevented EGFR OE and externally added rec. HB-EGF-mediated cell proliferation (Figure 3E,F). EGFR activation regulates MMP expression through transcriptional activation in various tumors [33]. Here we found that ALOC-EO inhibited EGFR-HB-EGF-mediated *MMP7/9*, and *ADAMA9* upregulation (Figure 3G). These data suggest that ALOC-EO blocks HB-EGF-EGFR-induced signaling and suppresses its downstream targets.

Chimeric and murine anti-EGF antibodies have proven effective in suppressing metastasis in a murine model for human melanoma. When EGFR was transiently silenced in B16F10 cells via the introduction of small interfering RNA (siRNA) or by adding the recombinant chimeric EGFR monoclonal antibody Cetuximab in the presence of ALOC-EO, melanoma growth was inhibited. Tumor cells could be completely blocked when ALOC-EO was added to cells treated with anti-EGFR or siRNA against EGFR (Supplementary Figure S1A). Because ALOC-EO showed an even stronger proliferation-suppressing effect when compared to cells where EGFR had been blocked, our data imply that, aside from EGFR-driven effects, ALOC-EO also mediates non-EGFR-mediated effects on cell proliferation. A similar pattern was observed when examining *MMP7* and *MMP9* expression of cultured cells (Supplementary Figure S1B,C). In summary, our data indicate that the antitumor effects in melanoma of ALOC-EO are driven by the blockade of EGFR signaling.

### 2.5. ALOC-EO Improves Chemosensitivity by Targeting EGFR Signaling

Resistance to chemotherapeutic agents is a concern for melanoma therapy. HB-EGF is an early response gene to chemotherapy and contributes to chemotherapy resistance [34]. Recent studies demonstrated that EGFR signaling contributes to a more malignant phenotype in melanoma-treated cells after chemotherapy [35,36]. 

**Figure 4:**
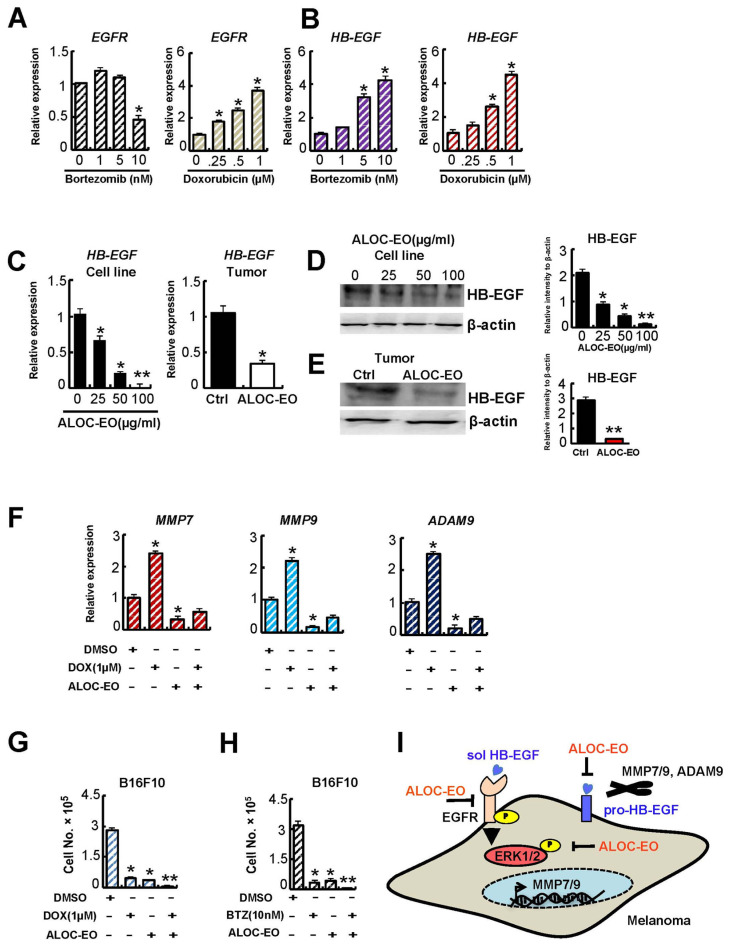
Chemotherapy-induced HB-EGF-EGFR upregulation is blocked by ALOC-EO and improves chemo-sensitivity. (**A**) Fold change expression of murine *HB-EGF* and (**B**) *EGFR* in B16F10 cells treated with indicated concentrations of the antitumor drugs bortezomib or doxorubicin, as a carrier by qPCR (A; n = 3/group). (**C**–**E**) HB-EGF expression was determined after ALOC-EO treatment for 24 h in B16F10 cells and tumor tissues retrieved from tumor-bearing mice treated with or without ALOC-EO at day 12 by qPCR (C; n = 3/group) by Western blotting analysis (**D**,**E**). Right panel: The histograms represent the signal intensity of the indicated protein bands in arbitrary units after normalization with the signal intensity of β-actin internal control for each sample. Values are means and SEM of two independent experiments. (**F**–**H**) B16F10 cells were treated with BTZ (**G**) or doxorubicin (**F**,**H**) at indicated concentration in the presence or absence of 100 µg/mL ALOC-EO. (**F**) Fold expression of *MMP7/9*, and *ADAM9* in cells treated with indicated drug combinations as determined by qPCR. (**G**,**H**) Viable cells were counted after 24 h, using the trypan blue exclusion assay (n = 6). The results of three independent experiments performed in triplicates are expressed as fold change according to 2-ΔΔCT method using β-actin as calibrator. Transcript levels were normalized to β-actin (n = 3/group). (**I**) Proposed mode of action for ALOC-EC in melanoma: ALOC-EO can block EGFR and ERK1/2 phosphorylation, leading to the reduced expression of MMP7/9 and ADAM9. Not shown in this study is that these proteases are known to enhance the shedding of the EGFR ligand HB-EGF. Abbreviations: ALOC-EO, Aloysia citrodora essential oil; EGFR, epidermal growth factor receptor; ERK1/2, extracellular signal-regulated kinase; MMP7, matrix metalloproteinase-7, matrix metalloproteinase9 (MMP9), a disintegrin and metalloproteinase domain-containing protein 9 (ADAM9). * *p* < 0.05; ** *p* < 0.01.

To investigate the possibility that melanoma cells modulate HB-EGF and/or EGFR expression to evade drug-induced cell death, we examined the expression of HB-EGF and EGFR after treatment with the anti-myelosuppressive drugs doxorubicin (DOX) and bortezomib (BTZ) [26,37]. The myelosuppressive drugs upregulated *HB-EGF* and *EGFR* in melanoma cells in a dose-dependent manner (Figure 4A,B). Next, we examined HB-EGF expression after ALOC-EO treatment. We found that ALOC-EO, but not carrier treatment, reduced HB-EGF expression in cultured melanoma cells (Figure 4C,D) and tumor extracts from ALOC-EO-treated tumor-bearing mice (Figure 4C,E).

Because ALOC-EO suppresses HB-EGF-EGFR signaling, we next determined ALOC-EOs’ ability to sensitize melanoma cells to chemotherapy. BTZ and DOX suppressed the EGFR downstream targets MMP7/9 and ADAM9 in a dose-dependent manner (Figure 4F, Supplementary Figure S1D), and cell proliferation (Figure 4G,H), which was further improved when ALOC-EO was added (Figure 4G,H). These data suggest that ALOC-EC can override myelosuppression-mediated drug resistance.

## 3. Discussion

Despite recent advances in the molecular, pathological, and biological understanding of melanoma, melanoma remains a devastating disease, especially in advanced disease stages due to drug resistance, demonstrating the urgency to identify novel effective treatments.

In the current study, we analyzed the effects of ALOC-EO on primary melanoma growth. We found that ALOC-EO inhibited the growth of skin cancer including melanoma cell lines in vitro, and showed its anti-proliferative effect using a murine melanoma model. Mechanistically, ALOC-EO inhibited EGFR signaling and prevented ligand (HB-EGF)-mediated melanoma cell proliferation. Our results showed that ALOC-EO blocked ERK1/2 phosphorylation and increased Bax in ALOC-EO-treated melanoma cells. Our data are in line with a study by Caudhary et al., who reported that the ALOC-EO compound geraniol inhibited the number of tumors in a murine skin tumorigenesis model by modulating cyclooxygenase-2 expression, Ras-ERK1/2 signaling, and upregulation of the pro-apoptotic BAX protein [29]. 

In cancer including melanoma, MMPs like MMP2/7/9 facilitate invasion/metastasis and participate as regulators of tumor cell proliferation and apoptosis [38,39,40,41]. These proteases degrade collagen type IV which is the major component of the basement membrane [42]. Earlier studies by Meierjohann et al. demonstrated that MMPs promoted melanoma migration and proliferation [43]. It was shown that EGFR signaling regulates both invadopodia formation and ECM degradation [44], and that EGFR inhibitors impaired MMP expression in melanoma cells [36]. In line with these reports, we could show that EGFR signaling blockade in part prevented MMP7/9 and ADAM9 expression. Given that these proteases are also important sheddases for HB-EGF, one of the main EGFR ligands, future studies should evaluate the potential of ALOC-EO to block MMP activation rather than transcriptional regulation.

Drugs inhibiting the activity of ERK1/ERK2 and downregulating the expression of MMP-9 can reduce invasiveness [45,46]. We showed that ALOC-EO suppressed ERK1/2 expression. This is of interest, as ERK1/2 mediates MAPK signals to cytoplasmatic and nuclear effectors. The anti-proliferative effect of geraniol, one of the compounds in ALOC-EO, was shown to reduce ERK1/2 expression on human colorectal adenocarcinoma Caco-2 cells and could be a potential candidate mediating the anti-melanoma effects observed with ALOC-EO in this study. Further studies will be necessary to test those compounds for melanoma cells. Another ALOC-EO compound is citral, which has been shown to block tumor progression in a two-stage skin-carcinogenesis model. Our results of the anti-EGFR effects of ALOC-EO are further substantiated by others demonstrating that ALOC-EO-treated fibroblasts show a decreased expression of tissue remodeling biomarkers collagen- and -III, plasminogen activator inhibitor-1 (PAI-1), and EGFR. Further studies will be necessary to determine which of the ALOC-EO compound(s) mediate the blockade of the HB-EGF-EGFR pathway in melanoma cells. We recently found that PAI-1 controls post-surgical adhesion through the EGF-HER1 axis. It will be interesting to demonstrate the potential of ALOC-EO for the treatment of postoperative adhesion after surgery [47].

A recent study demonstrated that stress-induced NRF2 binds to the ARE in the EGF promoter and leads to elevation of soluble EGF, while simultaneously blocking MITF activity, resulting in derepression of EGFR and TGFA [19]. This leads to EGFR activation. It will be interesting to determine whether ALOC-EC or its components modulate NRF2 expression.

Anti-EGFR monoclonal antibodies have been approved for the treatment of metastatic colorectal cancer [48] and head and neck cancer [49]. Erlotinib, which is an EGFR tyrosine kinase inhibitor, is FDA-approved for advanced/metastatic lung cancer [50]. However, the anti-EGFR targeting antibody Cetuximab could not influence the overall survival as shown in a study of second-line treatment of colorectal cancer [51].

Myelosuppressive drugs put therapeutic pressure on tumor cells, resulting in the disappearance of tumor clones or the upregulation of pro-survival signals that drive polyclonal-acquired drug resistance [52]. Melanoma or non-small cancer cells, among other cancer cells, acquire drug resistance by upregulating EGFR after BRAF exposure. Receptor tyrosine kinase signaling including HB-EGF-EGFR is upregulated in human SKmel-28 cells during the gain of resistance [53]. Other studies showed that six out of 16 melanoma tumors acquired EGFR expression after the development of resistance to BRAF or MEK inhibitors [54], and acquired resistance was found with the overexpression of signaling receptors like EGFR, PDGFRβ, or MET that reactivation the MAPK pathway. Following BRAF or MEK inhibitor treatment, melanoma tumors had acquired EGFR expression. Chemotherapy-induced EGFR activation is regulated by HB-EGF [34]. Accordingly, the further development of alternative agents based on EGFR signaling inhibition strategies are required to provide clinical benefit to patients with epithelial malignancies. Here, we showed that ALOC-EO could be one such therapeutic strategy to improve drug sensitivity in melanoma cells.

Oncogenic BRAF, NRAS, NF1 mutations can activate the tyrosine kinase receptor EGFR resulting in enhanced ERK1/2 phosphorylation in melanoma. The ERK1/2 pathway communicates signals from surface receptors like EGFR into the nucleus that is also linked to Raf mutations, known to occur in up to 90% of patients with melanoma. Activation of ERK1/2 signaling is activated as a cellular defense of human non-small cancer cells against the neutralizing antibody against EGFR (cetuximab) and fractionated irradiation treatment. These studies argue that the combined targeting of EGFR and ERK1/2 might be beneficial in these cancer types. Our data demonstrate that ALOC-EO inhibited ERK1/2 in melanoma cells, and provide another argument for ALOC-EO to be used as a co-treatment together with conventional antitumor drugs.

In conclusion, we demonstrated that ALOC-EC suppresses melanoma proliferation by blocking HB-EGF-EGFR signaling. As a result, the expression of proteases like MMP7/9 and ADAM9, which also can enhance HB-EGF shedding, was downregulated. We further showed that ALOC-EC blocked tumor cell migration. We propose that ALOC-EC might be effective in combination with myelosuppressive drugs to suppress drug-induced HB-EGF-EGFR expression. But further pharmacokinetic studies will be required to identify a safe drug delivery into affected tissues at relevant tissue concentrations. Altogether, our study highlights the potential of ALOC-EO as an anticancer agent that could be used in combination with selected drugs to improve treatment efficacy and overcome drug resistance.

## 4. Material and Methods

### 4.1. Chemicals and Cytokines

MTT (3-(4, 5)-dimethylthiazol (-z-y1)-3, 5-diphenytetrazoliumromide) and fluorescein isothiocyanate (FITC)–phalloidin was purchased from Sigma (St. Louis, MO, USA).

ERK1/2 inhibition was achieved with 10 uM PD98059 (MedChemExpress, Tokyo, Japan). The chemical is also a potent and selective MEK inhibitor with an IC_50_ of 5 µM by binding to the inactive form of MEK, thereby preventing the activation of MEK1 (IC_50_ of 2–7 µM) and MEK2 (IC_50_ of 50 µM) by upstream kinases. When DMSO was used for drug dilution, the final concentration of DMSO did not exceed 0.1% throughout the study. This DMSO concentration was found to not affect cell growth. Recombinant human HB-EGF was obtained from Peprotech (Cranbury, NJ, USA).

### 4.2. Generation of ALOC-EO

The generation of (ALOC-EO) from plants collected near Nablus, Palestine, was recently described in details Jaradat et al. [55] Briefly, dried leaf powder was suspended with 1 L of distilled water, and the EO was extracted using a Clevenger device operating at atmospheric pressure for 180 min at 100 °C. The obtained ALOC-EO was chemically dried using calcium carbonate and stored at 2 °C in the refrigerator until further use. The yield of the obtained EO was 1.31% *w*/*v*. The hydro-distilled ALOC-EO was qualitatively and quantitatively characterized utilizing gas chromatography-mass spectroscopy (GC-MS) technique. The ALOC-EO was dissolved in DMSO to a concentration of 200 µg/mL. Gas chromatographic (GC) analyses were completed using an HP 5890 series II gas chromatograph, equipped with a flame ionization detector and Perkin Elmer Elite-5-MS fused-silica capillary column (0.25 mm × 30 m, film thickness 0.25 µm) [55].

### 4.3. Cell Line and Cell Culture

The B16F10 melanoma cell lines (ATCC CRL-6475) were maintained in Dulbecco’s Modified Eagle Medium (DMEM, Gibco, Waltham, Massachusetts, USA) high glucose with L-glutamine, phenol red (Wako, Japan) medium containing 10% fetal bovine serum (FBS; HyClone, Logan, UT, USA), and 1% penicillin/streptomycin (P/S) (Nacalai Tesque Inc., Kyoto, Japan).

The human melanoma cell line SK-MEL-28 (ATCC HTB-72) and the human epidermoid carcinoma cell line A-431 (ATCC CRL-1555) were expanded in Eagle’s Minimum Essential Medium containing 10% FBS. Cells of the mouse stromal cell line MS5 and of the murine fibroblast cell line NIH/3T3 were cultured in Iscove’s Modified Dulbecco’s Medium with L-glutamine, and phenol red medium containing 10% FBS and 1%P/S. NIH/3T3 cells (ATCC CRL-1658) were maintained in DMEM. Murine embryonic endothelial progenitor cells (i.e., eEPCs (T17b cells) were cultured in DMEM/10%FBS.

### 4.4. Cell Proliferation Assay

Indicated cell lines were cultured in the presence or absence of ALOC-EO at indicated concentrations or other drugs. The cell viability was determined by trypan blue staining. In certain indicated experiments, the MTT assay was used to determine cell proliferation. After the drug exposure period, media was removed and cells were incubated with 20 uL 0.5% MTT in culture medium for an additional 4 h (Sigma, Burlington, MA, USA). The number of viable cells was directly proportional to the production of formazan, which was then solubilized with DMSO and measured spectrophotometrically at 570 nm. The experiments were repeated at least thrice.

### 4.5. Wound Healing (Scratch) Assay

B16-F10 motility was assessed using wound healing assay, as described in a previous report [56]. Cells were seeded into a six-well plate for nearly 80% confluence. When the cells were confluent, a 1-mL pipette tip spearhead was used to scratch/wound the six-well plate perpendicular to its bottom, with two lines of similar width per well. After the plate was washed gently with PBS three times, complete DMEM was again applied. The confluent cell monolayers were wounded by a sterile white pipette tip. The cells migrated into the cell-free space were counted using inverted microscopy; five randomly chosen fields were analyzed for each well. Three independent experiments were performed.

### 4.6. Cell Culture

SK-MEL-28, A-431, or B16F10 cells were seeded at 2 × 10^5^ cells/well in six-well plates (Thermo Fisher Scientific, Lafayette, CO, USA) in triplicate. NIC/3T3, T17b, or MS5 cell were seed at 1 × 10^5^ cells/well in six-well plates (Thermo Scientific) in triplicate. After 16 h overnight culture, cells were treated with or without indicated concentrations of ALOC-EO. Then, 24 h later, cells were counted or subjected to further analysis (qPCR or Western blotting). Cells were collected with TRIzol at indicated time points after treatment for RNA extraction.

5 × 10^4^ B16F10 cells were seeded in triplicate in six-well plates and incubated in the presence of specified treatments. Viable cells were counted at 24 and 48 h using the trypan blue dead cell exclusion dye (#207-17081, Wako). Treatment included the addition of human recombinant HB-EGF (100 ng/mL), PBS (control), or addition of Adriamycin or bortezomib and controls for 24 h alone or in combination with 100 uM ALOC-EO.

### 4.7. Cloning of Mouse EGF Receptor 

EGFR cloning: The mouse EGFR coding sequence was amplified by qPCR using brain tissue cDNA and the following primers were used 5-AAACTCGAGATGCGACCCTCAGGGACCGCGAGAAC-3 and 5-GGGGATATCTTAGATCCAAAAGGTCTTTAAGATC-3 to amplify mouse EGFR by genomic PCR, using PrimeSTAR polymerase (Takara, Tokyo, Japan). The purified fragment was inserted into the Xho I and EcoR V sites of the eukaryotic expression vector LV-EF-L3T4-IRES2-EGFP.

### 4.8. EGF Receptor Lentivirus Generation for Overexpression

Vesicular stomatitis virus glycoprotein-pseudotyped lentivirus was prepared as described in Kanegae et al. [57] via plasmid system (target vector, pMDL), and vesicular stomatitis virus glycoprotein envelope plasmid) by cotransfection of 293T cells with polyethylenimine (Takara, Shiga, Japan). The following plasmid was used as a mock: LV-EF-L3T4-IRES2-EGFP (kindly provided by Dr. Trono’s laboratory (Salk Institute, San Diego, CA, USA) and modified by Dr. Tomoyuki Yamaguchi (Institute of Medical Science, The University of Tokyo, Japan). The viral supernatant was collected 48 h later, cleared, and stored at −80 °C. Viral titration was performed using 293T cells.

### 4.9. siRNA-Based Gene Knockdown

For EGFR knockdown, cells were seeded at a 5x10^4^ cells/six-well concentration for 16 h before transfection. After 12 h, the transfection media was replaced with fresh media. Specific gene-targeting and control non-specific targeting small interfering ribonucleic acids (siRNAs) were obtained from Invitrogen (Thermo Fisher Scientific, Lafayette, CO, USA). siRNA targeting EGFR or a control sequence were transfected into cancer cells at a final concentration of 100 nM using Lipofectamine™ RNAiMAX Transfection Reagent. The siRNA target sequence for EGFR was 5′ -GGAUGUGAAGUGUGGCCAU-3′.

### 4.10. In Vivo Melanoma Model

Local tumor model: B16F10 cells were washed twice with PBS (90% viability, as determined by trypan blue exclusion) and then were inoculated on d 0 (1 × 10^6^/200 µL/mouse, s.c.) into mice, and tumor growth was observed. Mice were euthanized when they showed signs of severe pain, had bodyweight loss >20% when compared to the initial body weight, or appeared moribund. Tumor growth was monitored daily. Extracted tumors were weighed at d 12.

Treatment of local tumor model: ALOC-EO (100 mg/kg) or carrier (DMSO/PBS) were injected intraperitoneally daily for 5 days starting on day 5. 

Twelve days after subcutaneous and intravenous tumor cell inoculation, the animals were euthanized by cervical dislocation, and tumors and adjacent conjunctive tissues were removed. Pulmonary nodules were documented with a camera.

### 4.11. Immunoassay

The levels of cytokines were determined in plasma. Samples were measured using commercially available mouse-specific enzyme-linked immunosorbent assay (ELISA) kits for mouse MMP9 (R&D Systems, Minneapolis, MN, USA). Each sample was measured in duplicate.

### 4.12. Quantitative Reverse Transcriptase-Polymerase Chain Reaction (qPCR)

Total RNA was extracted from different cell lines using Trizol reagent and reverse-transcribed into cDNA using the PrimeScript RT Master Mix according to the manufacturer’s instructions. Total RNA was isolated from cultured cells or organ tissues using a Nucleospin RNA plus kit (Takara Bio, Otsu, Japan). The purity and concentration of the extracted RNA were analyzed by Nanodrop before complementary DNA (cDNA) generation according to the manufacturer’s protocols (Applied Biosystems, CA, USA) and was stored at −30 °C.

Each qPCR experiment was performed in triplicate and independently repeated two times. Gene expression was normalized for all qPCR results to β-actin mRNA expression unless otherwise mentioned. The respective forward and reverse primers used are:

qPCR Forward; Reverse
mMMP7 5′-TAATTGGCTTCGCAAGGAGA-′3; 5′-AAGGCATGACCTAGAGTGTTCC-′3
mMMP9 5′-AGACGACATAGACGGCATCC-′3; 5′-TCGGCTGTGGTTCAGTTGT-′3
mβ-actin 5′-CTAAGGCCAACCGTGAAAAG-′3; 5′-ACCAGAGGCATACAGGGACA-′3
mHB-EGF 5′-TCTTCTTGTCATCGTGGGACT-′3; 5′-CACGCCCAACTTCACTTTCT-′3
mEGFR 5′-ACGCATTCCTCCCTGTACCT-′3; 5′-GCAGGGGCTGATTGTGATAG-′3

### 4.13. Western Blotting Analysis 

Protein crude was recovered by Cell Lysis Buffer (10X; #9803, Cell Signaling, Danvers, Massachusetts, USA), and protease inhibitors (Complete Mini, Roche). Lysates were cleared by centrifugation at 10,000 rpm for 10 min at 4 °C. The total protein concentration of the supernatant was determined using a BSA protein (Bio-Rad Laboratories, Des Plaines, IL, USA). Cell lysates (2–50 μg proteins) were applied on 12% acrylamide gel, transferred to PVDF membrane (Millipore, Immobilon), and then probed with one of the following primary antibodies (all mouse IgG, 1 µg/mL) overnight at 4 °C: C-terminal cytoplasmic domain of HB-EGF of mouse origin (Santa Cruz Biotech, Santa Cruz, CA, USA, sc-1414), MMP7 (Santa Cruz Biotech, Santa Cruz, CA, USA, sc-515702), β-actin (Cell Signaling, #4967), total p44/42 MAPK (Erk1/2)(Cell Signaling, #9102), p-EGFR (Santa Cruz Biotech, sc-57545), MMP9 (Chemicom, AB19047)), ERK1/2 (Cell signaling, 4370S), BAX2 (Cell signaling, Danvers, MA, USA, #2772S). Membranes were stained with secondary antibody conjugated with horseradish peroxidase (Nichirei, rabbit-HRP, or goat-HRP), and developed with the ECL Plus detection system (Amersham Life Science, RPN2132) using image analyzer Image-Quant LAS4000 (GE Healthcare, Uppsala, Sweden).

### 4.14. Statistical Analysis

All experiments were performed at least three times. Data are shown as the mean ± standard error of the mean (SEM). *p* < 0.05 was considered to be significant. Multiple groups were compared using one-way ANOVA software and analysis of the two groups was done using the student *t*-test.

## Supplementary Materials

The following are available online at https://www.mdpi.com/article/10.3390/ijms22158151/s1.
