# Supplementary material for: Aloysia Citrodora Essential Oil Inhibits Melanoma Cell Growth and Migration by Targeting HB-EGF-EGFR Signaling
Source: Int J Mol Sci. 2021 Jul 29;22(15):8151. doi: 10.3390/ijms22158151 (PMC8347434; doi:10.3390/ijms22158151)
Supplement: Supplementary file 1 [file ijms-22-08151-s001.zip › ijms-1312404-supplementary.pdf]

Supplementary Table S1. Chemical components of ALOC-EOs collected from the Nablus region, Palestine.

Scientific names	Names of EOs	Formula	Chemical structure	RT	RI_{calc}	RT_{Litr}	% of EOs
2-methyl-5-propan-2-ylbicyclo[3.1.0]hex-2-ene	α-Thujene	$C_{10}H_{16}$	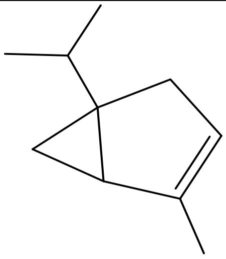	8.71	821	821	0.27
4-methyl-1-propan-2-ylbicyclo[3.1.0]hex-2-ene	β-Thujene	$C_{10}H_{16}$	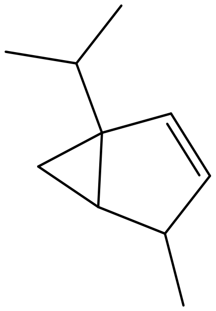	10.51	874	875	0.83
1-Octen-3-ol	Morillol	$C_8H_{16}O$	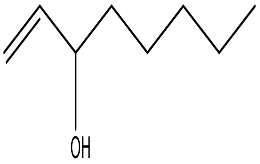	10.79	712	712	0.18
2-(5-ethenyl-5-methyloxolan-2-yl)propan-1-ol	Lilac alcohol C	$C_{10}H_{18}O_2$	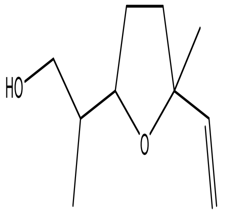	10.96	597	598	1.06
(4*R*)-1-methyl-4-prop-1-en-2-ylcyclohexene	**D-Limonene**	$C_{10}H_{16}$	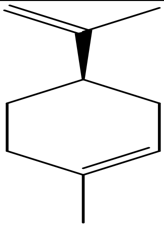	12.71	895	894	14.84
1,3,3-trimethyl-2-oxabicyclo[2.2.2]octane	Eucalyptol	$C_{10}H_{18}O$	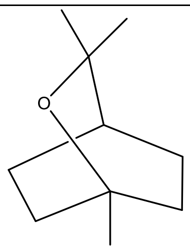	12.83	826	827	4.48

3,7-dimethylocta-1,3,6-triene	β-Ocimene	$C_{10}H_{16}$	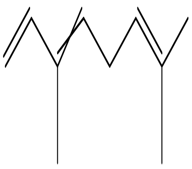	13.45	873	872	0.92
(1*S*,5*S*)-6,6-dimethyl-2-methylidenebicyclo[3.1.1]heptane	β-Pinene	$C_{10}H_{16}$	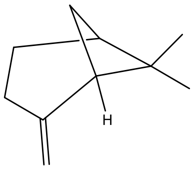	15.63	902	902	0.31
2-(4-methylcyclohex-3-en-1-yl)propan-2-ol	α-Terpineol	$C_{10}H_{18}O$	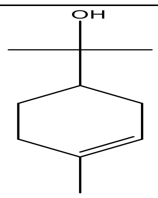	19.29	903	902	1.99
2,6-Octadienal, 3,7-dimethyl-	**Neral**	$C_{10}H_{16}O$	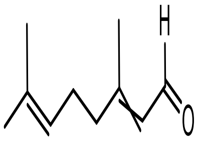	20.9	723	722	20.03
(2*E*)-3,7-dimethylocta-2,6-dienal	**Geranial**	$C_{10}H_{16}O$	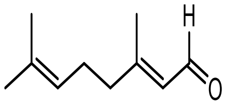	21.97	838	838	26.61
(1*S*,6*S*,7*S*,8*S*)-1,3-dimethyl-8-propan-2-yltricyclo[4.4.0.0^{2,7}]dec-3-ene	Copaene	$C_{15}H_{24}$	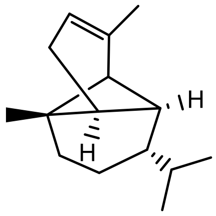	25.59	832	832	0.46
7-methyl-3-methylideneocta-1,6-diene	β-Myrcene	$C_{10}H_{16}$	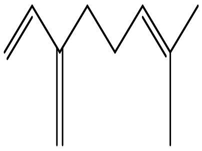	25.68	738	737	1.14
1-methyl-5-methylidene-8-propan-2-yltricyclo[5.3.0.0^{2,6}]decane	β-Bourbonene	$C_{15}H_{24}$	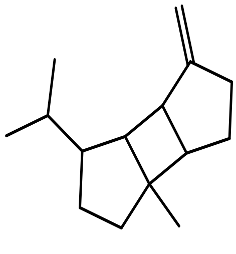	25.87	859	859	0.42

2-methyl-5-(6-methylhept-5-en-2-yl)cyclohexa-1,3-diene	α-Zingiberene	$C_{15}H_{24}$	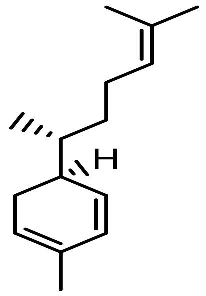	26.93	789	791	0.51
(1*R*,4*E*,9*S*)-4,11,11-trimethyl-8-methylidenebicyclo[7.2.0]undec-4-ene	β-Caryophyllene	$C_{15}H_{24}$	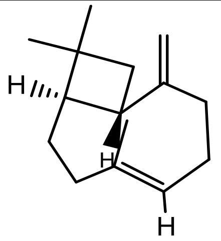	27.04	890	890	3.33
(1*S*,6*S*,7*S*,8*S*)-1-methyl-3-methylidene-8-propan-2-yltricyclo[4.4.0.0^{2,7}]decane	β-Copaene	$C_{15}H_{24}$	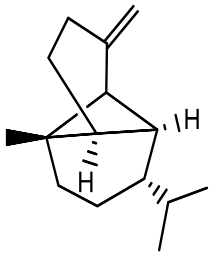	27.18	716	717	0.16
1,1,7-trimethyl-4-methylidene-2,3,4*a*,5,6,7,7*a*,7*b*-octahydro-1*aH*-cyclopropa[e]azulene	Aromadendrene	$C_{15}H_{24}$	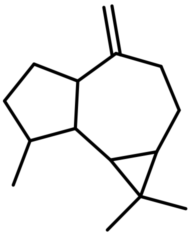	28.33	825	825	0.43
(1*S*,5*S*,6*R*)-2,6-dimethyl-6-(4-methylpent-3-enyl)bicyclo[3.1.1]hept-2-ene	trans-α-Bergamotene	$C_{15}H_{24}$	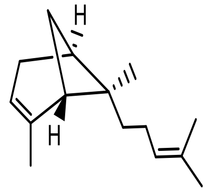	28.6	727	726	0.24
1-methyl-4-(6-methylhept-5-en-2-yl)benzene	α-Curcumene	$C_{15}H_{22}$	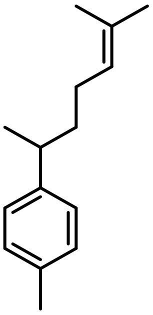	28.96	896	896	13.97

(3*R*,4*R*)-4-ethenyl-4-methyl-1-propan-2-yl-3-prop-1-en-2-ylcyclohexene	δ-Elemene	$C_{15}H_{24}$	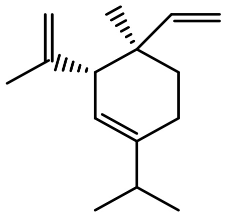	29.45	781	781	0.62
(3*E*,6*E*)-3,7,11-trimethyldodeca-1,3,6,10-tetraene	α-Farnesene	$C_{15}H_{24}$	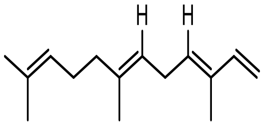	31.39	772	772	0.96
(1*aR*,4*aR*,7*S*,7*aR*,7*bR*)-1,1,7-trimethyl-4-methylidene-1*a*,2,3,4*a*,5,6,7*a*,7*b*-octahydrocyclopropa[h]azulen-7-ol	Spathulenol	$C_{15}H_{24}O$	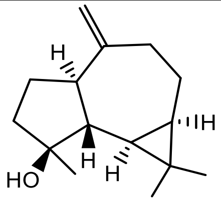	31.91	735	735	1.72
4,10,11,11-tetramethyltricyclo[5.3.1.0^{1,5}]undecane	Patchoulane	$C_{15}H_{26}$	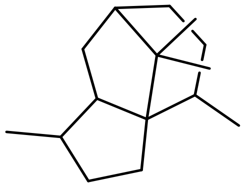	32.07	851	851	4.53
Total							100
**Phytochemical classification of *A. citrodora* EO**							
Hydrocarbon monoterpene							18.31
Oxygenated monoterpenoid							54.16
Hydrocarbon sesquiterpene							25.63
Oxygenated sesquiterpenoid							1.72
Fatty alcohol							0.18
Total							100

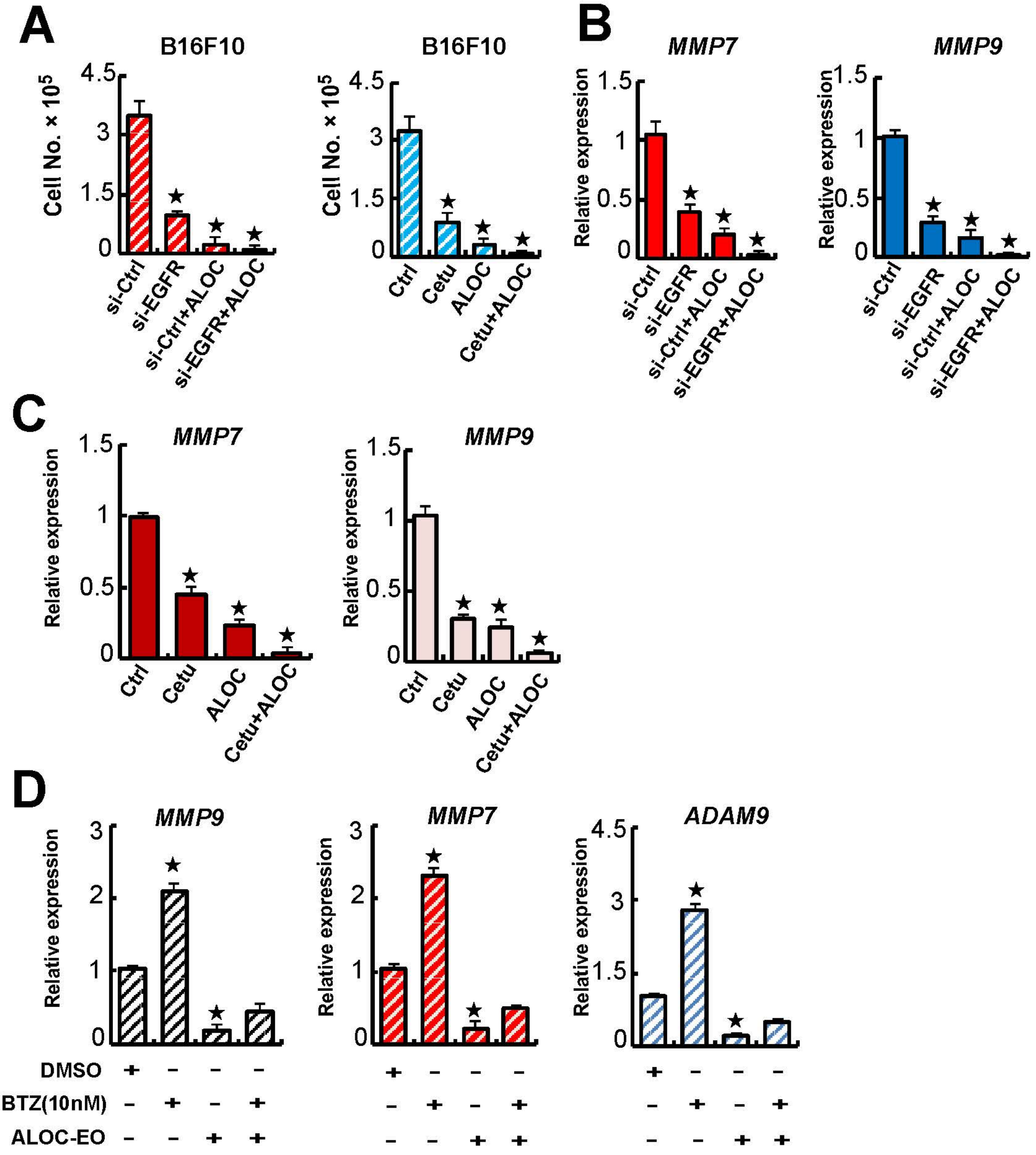

Supplementary Figure S1. ALOC-EC inhibits myelosuppression-induced protease expression. (A-C) B16F10 EGFR control cells or cells where EGFR had been blocked either by gene silencing with siRNA (si-EGFR; left panel) or due to the addition of neutralizing antibodies against EGFR (Cetuxamib) were cultured in the presence or absence of ALOC-EO (n=3/group). (A) Proliferation using a cell count was done after 24 h in culture. (B and C) Cells were collected 24 h after the addition of ALOC-EO as indicated, the expression of MMP7 and MMP9 were deter-mined by qPCR. Presented is relative gene expression. Transcript levels were normalized to b-actin. (D) B16F10 cells

were treated with BTZ (10 nM) in the presence or absence of 100 ug/ml ALOC-EO. Fold expression of MMP7/9, ADAM9, in cells treated with indicated drug combinations as determined by qPCR. (G and H) Viable cells were counted after 24 h using the try-pan blue exclusion assay (n=6). The results of three independent experiments performed in triplicate are expressed as fold change according to 2-ΔΔCT method using b-actin as calibrator. Transcript levels were normalized to b-actin (n=3/group).
